# Outbreaks of histoplasmosis: The spores set sail

**DOI:** 10.1371/journal.ppat.1007213

**Published:** 2018-09-13

**Authors:** George S. Deepe

**Affiliations:** Division of Infectious Diseases, Department of Medicine, University of Cincinnati College of Medicine, Cincinnati, Ohio; McGill University, CANADA

Human infection with the pathogenic fungus *Histoplasma capsulatum* produces diverse clinical manifestations, ranging from an influenza-like illness to a cavitary lung disease to life-threatening dissemination affecting multiple major organ systems [1, 2]. Long considered to be an infection endemic to the midwestern and southeastern United States and Central and South America [3, 4], new epidemiological reports indicate that other areas of the world, and the US for that matter, have indigenous cases. There is increasing recognition of histoplasmosis in China, particularly along the Yangtze River [5, 6], and numerous case reports are emerging from India [7]. In the US, the recent description of cases from Montana suggests that *H*. *capsulatum* is wider spread than originally considered.

## Biology and natural history of *H*. *capsulatum*

This fungus is considered to be dimorphic; it grows both as a mold and a yeast. The phase depends on temperature. At 25 °C, *H*. *capsulatum* exists as a mold and generates two types of conidia or spores-macroconidia and microconidia. These forms are asexual ovoid structures produced at the tips of hyphae. The portal of entry is the lung, and microconidia are considered to be the form that reaches the alveoli and terminal bronchioles of the lung because of their small size (2–5 μm). Exposure of the mold phase to 37 °C induces an orderly change in gene expression, driving conversion of spores into yeast cells that are typically 2–4 μm in diameter [8]. It is this morphotype that causes nearly all the pathology associated with histoplasmosis [3]. Since yeast cells are not transmissible from human to human, they do not promulgate transmission of the fungus in outbreaks.

## The role of bird and bat droppings

The habitat of *H*. *capsulatum* is soil laden with bird excreta or bat guano. Contact with the fungus usually requires disturbing the soil, thus aerosolizing spores. Droppings from several avian species have been implicated in supporting the growth of the fungus; these include starlings, blackbirds, pigeons, and, less commonly, oilbirds (found in South America) and grackles [9]. It was not until the 1940s that *H*. *capsulatum* was recovered from the soil, thus establishing incontrovertibly that infection is acquired from the environment. The fungus was isolated from soil containing chicken excreta surrounding a chicken coop [9]. The organism is usually found within 20 cm of the ground surface. It thrives in acidic soil rich in nitrogen. This compound is more freely accessible in decomposing rather than fresh excreta. In heavily infested soils, the number of *H*. *capsulatum* particles has been estimated to reach 10^5^ per gram of soil [10, 11]. Of note, conidia (or spores) have been identified in soil, and this finding documents the presence of this morphotype in nature [12].

Despite numerous attempts to culture the fungus from environmental reservoirs, *H*. *capsulatum* has been notoriously difficult to isolate directly from the soil, although occasionally using a mineral oil flotation process has achieved success. Rather, the simplest and perhaps most reliable method is to inoculate mice intraperitoneally with an emulsion of the soil, wait approximately 15 to 30 days, and culture several organs, including liver, spleen, and lungs, for the presence of the fungus [13]. The mouse mounts sufficient host defenses to eradicate the saprophytes found in the soil. Thus, endemic niches are frequently identified by outbreaks in humans or domestic animals, such as dogs, rather than by recovering fungus from environmental sites.

What is it about bird excreta or bat guano that fosters the presence of *H*. *capsulatum*? To this day, the answer to this question remains elusive. Attempts have been made in the past, but this work has long been neglected. What we do know is that a carbohydrate from chicken excreta, not otherwise identified, produced excellent growth and sporulation of *H*. *capsulatum*. Other constituents in excreta that promote growth of the fungus include salts, particularly phosphate, which is enriched in excreta compared to the surrounding soil, and nitrogen. The nitrogenous substances that fuel growth remain unknown, although urea has been suggested but not definitively documented [9, 11].

## *Histoplasma* outbreaks

Numerous reports of epidemics or outbreaks of histoplasmosis in the US and Canada have been described over the years, dating back to the late 1930s [14–16]. An outbreak is defined as involving at least two cases. In 1963, 31 cases of histoplasmosis were identified in Montreal along the St. Lawrence river valley. Although no specific source was detected, the authors noted that the city had been in a construction boom during this period. Perhaps the most ironic episode of multiple infections transpired on Earth Day in 1970. A large number of junior high school students and faculty in Delaware, Ohio were infected after clearing a school courtyard that had been a bird roost [17].

The outbreak that developed in Montreal in the 1960s is an exception to the rule that, prior to the 1970s, most outbreaks developed in rural areas, as might be expected for an infection associated with bird or bat excreta. Given that many of these episodes originally were reported in rural areas, the number of individuals infected often has been fewer than 100. Since the 1970s, the setting for epidemics has shifted from rural to urban [14]. With a shift to a more urban exposure, it is not unexpected that the number of infected individuals exceeds that found in rural areas. The extreme examples of urban epidemics are the two in Indianapolis, Indiana that occurred in the late ‘70s and early ‘80s [18–21]. In the first outbreak, more than 100,000 residents of Indianapolis were infected. It was associated with razing an old amusement park and construction of a tennis stadium in the downtown area. The epidemic persisted for one year and likely had windborne spread, as is more often observed with the spread of *Coccidioides* sp. The second outbreak resulted from construction of a new natatorium on the campus of Indiana University–Purdue University Indianapolis (IUPUI). In total, approximately 200,000 individuals were infected as a consequence of the two outbreaks.

Another important element of these two episodes is the detrimental impact *H*. *capsulatum* had on the health of Indianapolis residents. Although outbreaks of histoplasmosis typically do not cause a high degree of mortality or morbidity and much of the disease is self-limited, these two were accompanied by more than 300 hospitalizations and at least 15 deaths. There were more than 40 cases of disseminated histoplasmosis. It is important to note that these outbreaks preceded the AIDS epidemic and the introduction of new biologicals such as tumor necrosis factor-α antagonists that are major risk factors for disseminated histoplasmosis. The reason for the devastating impact is not clear, but the risk factors that were identified included ages greater than 54 and immunosuppression. The scope of these two epidemics establish the importance of the urban setting, wind, and immune status when considering the impact of histoplasmosis on a population.

Indianapolis did suffer another epidemic in the late ‘80s to the early ‘90s. However, this time, many of the cases developed in AIDS patients. This outbreak was not traced to a specific demolition or construction site. Rather, it might have been foci of *H*. *capsulatum* in the city and its neighborhoods that caused this increase in cases [22]. The major difference was that a highly susceptible population was now exposed, thus bringing the problem to medical attention earlier.

## A spelunker’s concern

An often neglected but important source of *Histoplasma* outbreaks is a cave [23]. Bats have been associated with the development of histoplasmosis for many years, and seasoned spelunkers are aware of the risk. Bats harbor the fungus, and it has been isolated from their feces and other tissues. However, the caves do not necessarily have to be in the traditionally endemic regions since cave-associated histoplasmosis has been reported from Florida, South Africa, Tanzania, Cyprus, Australia, and Zimbabwe.

Bats are not only found in caves but can be present in tunnels. A recent outbreak in the Dominican Republic highlighted this association [24]. A dam had five associated tunnels in which bats had been roosting, and a company was contracted to remove the guano, which reached up to one meter deep from the tunnels. Subsequently, all 36 workers became ill, and their illness was initially misdiagnosed. Of those exposed, >90% received antifungal therapy, indicating the severity of disease and likely the number of spores in the bat guano.

## The indoors and acquisition of histoplasmosis

Not all the exposures to *H*. *capsulatum* require individuals to be in proximity to areas that are being excavated or where soil is disrupted. One outbreak occurred in a hotel in Acapulco, Mexico in which cleaning of air ducts and the use of stairwells was associated with the spread of spores that caused illness in 21 students who were vacationing over spring break [25]. Another outbreak was attributed to the dissemination of spores via the air handling system at the University of Texas Southwestern Medical School [26]. The likely source was a bird sanctuary near campus. The transmission was noted to be strictly indoors, and cases were more frequent in the upper floors. The air handling system was not constructed to remove small particles such as *H*. *capsulatum* spores. Other outbreaks related to air handling systems include one in which room air conditioners spread spores that had been swept off a roof of the building.

## Summary and conclusions

Infection with *H*. *capsulatum* is not a reportable disease, and therefore, tracking the number of cases is a difficult task. Usually, histoplasmosis comes to our attention when there are outbreaks, whether they involve a few or a few thousand individuals. Once considered a rural disease of the Midwest and Southeast, the pattern of outbreaks has shifted to involve urban areas. Concomitantly, recognition of the disease as one of the Americas is simply no longer true. The geographic extent of the disease has broadened considerably, with an increasing number of reports from Asia. Occupational workers must be provided with the appropriate knowledge regarding the risks when they are tasked with environmental remediation for a site that potentially contains *H*. *capsulatum* spores. Unfortunately, no safe soil disinfectant is available that will kill spores; hence, the best protection for workers is the proper protective equipment and wetting of the soil to minimize aerosols.
